# Prevalence of Overweight and Obesity in the Middle-age Population: A Priority for the Health System

**Published:** 2017-06

**Authors:** Mehdi KHABAZKHOOB, Mohammad Hassan EMAMIAN, Hassan HASHEMI, Mohammad SHARIATI, Akbar FOTOUHI

**Affiliations:** 1. Dept. of Medical Surgical Nursing, School of Nursing and Midwifery, Shahid Beheshti University of Medical Sciences, Tehran, Iran; 2. Center for Health Related Social and Behavioral Sciences Research, Shahroud University of Medical Sciences, Shahroud, Iran; 3. Noor Research Center for Ophthalmic Epidemiology, Noor Eye Hospital, Tehran, Iran; 4. Dept. of Community Medicine, School of Medicine, Tehran University of Medical Sciences, Tehran, Iran; 5. Dept. of Epidemiology and Biostatistics, School of Public Health, Tehran University of Medical Sciences, Tehran, Iran

**Keywords:** Overweight, Obesity, Middle east, Prevalence

## Abstract

**Background:**

The aim of this study was to determine the prevalence of overweight and obesity and their associated factors in the middle-aged population of Shahroud, North of Iran.

**Methods:**

In a population-based cross-sectional study with random cluster sampling, 300 clusters of Shahroud (north of Iran) were randomly selected from the 40–64 yr old population of the city, during 2009 and 2010. Upon enrollment, participants were weighed on digital scales and their heights were measured under standard conditions. Overweight and obesity were defined as a body mass index (BMI) of 25–29.9 kg/m^2^ and a BMI ≥30 kg/m^2^, respectively.

**Results:**

Of the 6311 selected people, 5190 people (82.2%) participated in the study. Their mean age was 50.9±6.2 yr, and 2977 of them were women (57.4%). Age and gender standardized mean BMI in the study population were 28.1 kg/m^2^ (95% CI: 27.9–28.2). Age and gender-standardized prevalence of BMI≥25 was 74.4% (95% CI: 73.0–75.8); 43.4% (95% CI 44.8–42.0) were overweight and 31.0% (95% CI 32.5–29.6) were obese. Overweight, Obesity and a BMI ≥25 prevalence’s were significantly higher in women (*P*<0.001). In the multiple logistic regression models, female gender and higher economic status were significantly correlated with BMI≥25. In addition, people over the age of 54 yr were more likely to have BMI≥25 than those in the 40–44 yr age range (*P*<0.001).

**Conclusion:**

The prevalence of overweight and obesity in the present study, especially in women, was higher than that reported from previous studies in Iran. Considering prevention, programs for overweight and obesity-related are suggested as a priority for the health system in this population.

## Introduction

According to the latest report by WHO, over-weight and obesity are one of the five primary causes of death worldwide, and in medium to high-income countries, these conditions are considered among the top three risk factors for mortality (1). On the other hand, the silent epidemic of overweight and obesity is spreading globally, and compared to 1980, the prevalence of obesity has more than doubled in the world (2).

Obesity and diabetes are responsible for an annual 2.8 million deaths among adults around the world (3). Moreover, in the United States, 5% to 10% of the health-related expenditure is spent on issues related to overweight and obesity (4). According to data available from the WHO, 61.9% of the over 20 yr old population in Americas and 54.8% of the population in Europe has a body mass index (BMI) ≥ 25 (5). After the US and Europe, the highest prevalence of overweight and obesity is in the Eastern Mediterranean region (6).

One of the most important reports on the status of obesity in Iran shows that during the years 1999–2007, the prevalence of obesity in people 25 to 64 yr of age increased from 13.6% to 22.3% (7), and the considerable increase in the mean BMI in this study is disturbing.

According to the latest WHO data on the status of overweight and obesity in Iran in 2008, more than 50% of the over 20 yr old population in Iran has BMI≥ 25, and 21.6% of Iranians over 20 yr old are obese (BMI≥30) (3,5). Given that over-weight and obesity are responsible for 44% of the burden of diabetes, 23% of ischemic heart disease, and 7%–14% of the burden of cancer (1), the rapid growth of obesity has led to concerns about this disease in future years.

Due to the growth in obesity over the past decade, more studies need to be conducted in different regions in Iran, so that potential outbreaks can be detected in early stages. Although the incidence of obesity is higher and increases faster after the fourth decade of life compared to younger ages, breakdown of our information by age, especially people 40 yr and older, is limited. The purpose of this report was to show the prevalence of overweight and obesity in Shahroud among 40 to 64 yr old adults.

## Materials and Methods

The present study is the first phase of the Shahroud Eye Cohort Study conducted cross-sectional from Feb 2009 to Jan 2010. Details of its protocol have been published elsewhere (8) and given here in brief.

The target population was the 40–64 yr old population of the northern Iranian city of Shahroud which has a population of 133835 based on the 2006 census. Sampling was done using the multistage cluster sampling method. Three hundred clusters were selected randomly from among the 9 strata (health centers), proportionate to the population of each stratum.

The first household in each stratum was selected using a random systematic approach and household record numbers available at health care centers. After identifying the first household, we approached the adjacent households, moving in a clockwise direction, until the target number of people was reached. Since every 40–64 yr, old household member was entered into the study, each cluster included at least 20 persons, depending on the number of eligible people in the last household. After being enrolled, informed consent was obtained from all participants before commencing examinations.

### Weight and height measurements

Weight measurements were done using digital scales tested first every day using 5 and 20 kg reference weights, and they were replaced if not immediately repairable. Height measurements were done without shoes, while participants gazed forward and held their heads straight up.

The measurement device was tested against a standard every week, and necessary adjustments were made in case of discrepancy. After weight and height measurements, their BMI was calculated using the following formula: weight (in kilograms) divided by height (in meters) squared.

In this study, we used the same BMI categories defined in previous studies. Overweight and obesity were defined as a BMI of 25–29.9 and BMI≥30, respectively. To examine the degree of obesity, these cases were further categorized into other groups. Obesity was divided into three categories; grade 1 for BMI of 30–34.9, grade 2 for BMI of 35–39.9, and grade 3 for BMI≥40. To examine economic inequality, we used the principal component analysis (PCA) method, and according to information related to 11 household items, we found that the greatest contribution related to owning an in-house bath, microwave, and dishwasher. These three factors accounted for 51.77% of the variance in the data. Then, an asset index variable was constructed based on giving weight to the first PCA factor, and this new variable was then divided into 3 tertiles. The first tertile reflected the group with high living standards, and the third tertile was considered the group with low living standards.

### Statistical analysis

In this study, the prevalence of overweight and obesity is given in percentages and their 95% confidence intervals (CI). Age and gender standardized rates of overweight and obesity were calculated based on the 2006 Shahroud population data. To calculate standard errors, the design effect of cluster sampling was accounted for. In addition, to show the BMI status, the mean, standard deviation, and 95% confidence intervals were determined. To assess the relationship between over-weight and obesity with potential risk factors, we used simple and multiple logistic regression models. In addition, assessment of the relationship between BMI and other studied parameters was done using linear regression. In the assessment of relationships with overweight and obesity, we used people with BMI<25 as the control group.

### Ethical approval

The Ethics Committee of Shahroud University of Medical Sciences approved the study protocol, conducted in accordance with the tenets of the Helsinki Declaration. All participants signed a written informed consent.

## Results

In this study, 5190 (82.2%) of the 6311 selected people participated in the study. Mean age of the participants was 50.9 ±6.2 yr, and 2977 (57.4%) of them were women.

### Body mass index

Age and gender standardized mean BMI in this study were 28.1 kg/m^2^ (95% CI: 27.9–28.2). The crude value for this mean was 28.4 kg/m^2^ (95% CI: 28.2–28.5). Mean BMI was 29.7 kg/m^2^ (95% CI: 29.5–29.8) in women and 26.6 kg/m^2^ (95% CI: 26.5–26.8) in men (*P*<0.001). Mean BMI by age and gender is shown in Table 1.

**Table 1:** Mean body mass index (BMI) and prevalence of overweight, obesity, and BMI≥25 in the studied population by age and gender

**All**		**BMI (kg/m^2^)**	**Overweight: BMI=25–29.9**	**Obese: BMI>=30**	**BMI>=25**
		Mean (95%CI)	% (95%CI)	% (95%CI)	% (95%CI)
40–44	960	28.5 (28.2–28.8)	43.4 (40.7–46.2)	33.4(30.5–36.4)	76.9(74.2–79.5)
45–49	1390	28.3 (28.0–28.6)	42.4 (39.8–44.9)	33.7(31.1–36.4)	76.1(73.7–78.5)
50–54	1285	28.3 (28.1–28.6)	41.3 (38.5–44.2)	33.1(30.4–35.8)	74.4(71.9–76.9)
55–59	954	28.3 (28.0–28.6)	42.3 (39.1–45.6)	35.0(32.0–38.0)	77.4(74.8–79.9)
60–64	601	28.4 (28.0–28.7)	46.1 (42.2–50.0)	31.6(27.9–35.4)	77.7(74.3–81.2)
Total	5190	28.4 (28.2–28.5)	42.7 (41.4–44.1)	33.5(32.1–34.9)	76.2(75.1–77.4)
**Age-standardized**		28.1 (27.9–28.2)	43.4 (42.0–44.8)	31.0(29.6–32.5)	74.4(73.0–75.8)
**Male**					
40–44	326	26.5 (26–26.9)	47.2 (42.2–52.3)	16.6(12.6–20.6)	63.8(58.9–68.7)
45–49	569	26.7 (26.3–27.0)	46.0 (41.9–50.2)	19.5(16.3–22.7)	65.6(61.3–69.8)
50–54	569	26.5 (26.1–26.8)	42.0 (37.8–46.2)	19.3(15.9–22.7)	61.3(57.4–65.3)
55–59	457	26.8 (26.5–27.2)	44.6 (40.0–49.3)	22.8(19.0–26.5)	67.4(63.1–71.7)
60–64	292	26.9 (26.4–27.3)	54.1 (48.4–59.8)	18.2(13.7–22.6)	72.3(66.9–77.6)
**Total**	2213	26.6 (26.5–26.8)	46.0 (43.8–48.1)	19.5(17.7–21.3)	65.5(63.5–67.4)
**Female**					
40–44	634	29.6 (29.2–30.0)	41.5(37.9–45.0)	42.1(38.3–45.9)	83.6(80.7–86.5)
45–49	821	29.5 (29.1–29.8)	39.8(36.5–43.1)	43.6(40.0–47.2)	83.4(80.8–86.1)
50–54	716	29.8 (29.5–30.2)	40.8(37.0–44.6)	44.0 (40.4–47.6)	84.8(82.1–87.5)
55–59	497	29.7 (29.3–30.1)	40.2(35.9–44.6)	46.3(42.1–50.4)	86.5(83.8–89.3)
60–64	309	29.8 (29.2–30.3)	38.5(33.4–43.6)	44.3(38.9–49.8)	82.8(78.8–86.9)
**Total**	2977	29.7(29.5–29.8)	40.3(38.6–42.0)	43.9(42.1–45.7)	84.2(83.0–85.4)

CI: confidence interval

In an analysis of variance model, after adjusting for gender, mean BMI had no significant difference in different age groups (*P*=0.666). Mean BMI in low, middle, and high economic status groups was 28.5 ±5.2 kg/m^2^, 28.5 ±4.8 kg/m^2^, and 28.2 ±4.5 kg/m^2^, respectively; the BMI in the middle economic class was significantly higher than in the high economic class (*P*=0.026). Nonetheless, after adjusting for education, there were no significant differences among economic groups in terms of BMI.

### Overweight

The prevalence of overweight by age and gender in the studied population is presented in Table 1. The prevalence of overweight in the studied sample was 42.7% (95% CI: 41.1–44.1), 46.0% in men and 40.3% in women. The odds of being overweight compared to the BMI<25 groups, was higher for men than women (OR=1.93; 95% CI: 1.70–2.18). The prevalence of overweight was significantly higher only in the 60–64 yr old group compared to the 40–44 yr old age group (*P*=0.016). The prevalence of overweight was 35.8% among the illiterate, and it increased up to 50% with higher levels of education. After adjusting for age and gender, the prevalence of over-weight, compared to the BMI<25 group, significantly increased with education (OR=1.02 for every year of education; 95% CI: 1.01–1.03). There was no significant association between the prevalence of overweight and marital status. The prevalence of overweight in high, middle, and low economic status groups was 46.1%, 42.8%, and 38.9%, respectively; according to our findings, the prevalence of overweight in the middle economic group (OR=1.2; 95% CI: 0.99–1.5) was higher than the low economic group, nonetheless, when compared to the BMI<25 group, the prevalence of overweight was significantly higher in the high economic group than the low economic group (OR=1.3; 95% CI 1.1–1.6). In a multiple logistic regression models, female gender, age between 60 and 64 yr, and middle and high economic status were significantly correlated with overweight.

### Obesity

As demonstrated in Table 1, the prevalence of obesity in the present study was 33.5% (95% CI: 32.1–34.9), 43.9% and 19.5% in women and men, respectively. The odds of obesity was 5.06 times higher for women (*P*<0.001). Prevalence of obesity grade 1, 2, and 3 were 25.6%, 6.2%, and 1.7%, respectively. There were no significant differences in prevalence’s of obesity among age groups (*P*=0.643).

As shown in Table 2, compared to the BMI<25 group, the prevalence of obesity was higher among those with less education. Logistic regression revealed a higher prevalence of obesity among the illiterate compared to those with college education (*P*<0.001). The prevalence of obesity was not significantly associated with the economic status in the simple logistic regression model. As Table 3 shows the results of logistic regression, the odds of obesity was significantly less among single people compared to the divorced and widowed (*P*<0.001). The multiple models revealed that female gender and age between 55 and 59 yr significantly associated with obesity.

**Table 2:** Prevalence of overweight and obesity by level of education, economic status, and marital status

**Variable**		**Overweight: BMI = 25–29.9**	**Obese: BMI>=30**	**BMI>=25**
	n	% (95%CI)	% (95%CI)	% (95%CI)
**Educational level**				
Illiterate	427	35.8 (31.0–40.7)	39.6(34.6–44.6)	75.4 (71.3–79.5)
Primary school	2595	41.5 (39.6–43.4)	32.7(30.6–34.8)	74.2 (72.3–76.2)
Middle School	481	43.2 (38.1–48.2)	31.9(27.6–36.1)	75.0 (70.2–79.8)
High school	1146	46.5 (43.5–49.4)	30.2(27.3–33.2)	76.7 (74.0–79.4)
College	541	50.0 (45.3–54.6)	19.8(16.2–23.3)	69.8 (65.6–73.9)
**Economic status**				
Low	1373	38.9 (36.0–41.8)	33.7 (30.9–36.4)	72.5 (69.8–75.3)
Medium	1433	42.8 (40.4–45.3)	32.4 (29.9–34.8)	75.2 (72.7–77.7)
High	2376	46.1 (44–48.1)	28.9 (27.0–30.9)	75.0 (73.0–77.0)
**Marital status**				
Single	67	42.4 (28.8–55.9)	24.0 (12.2–35.8)	66.3 (52.8–79.9)
Married	4796	43.6 (42.1–45.1)	30.6 (29.1–32.1)	74.2 (72.8–75.6)
Widowed	291	39.2(33.9–44.6)	41.1 (35.6–46.7)	80.3 (76–84.7)
Divorced	36	43.0 (26.1–59.8)	42.5 (24.1–60.8)	85.4 (73.8–97)

CI: confidence interval

**Table 3:** Simple and multiple logistic regression analysis between obesity, BMI≥25, and independent variables

**Variable**	**Obesity**		**BMI>=25 (Overweight + obesity)**	
	Simple OR^*^ (95%CI)	Multiple OR^*^ (95%CI)	Simple OR^**^ (95%CI)	Multiple OR^**^ (95%CI)
**Sex (Female=1, Male=0)**	5.06 (4.31–5.94)	5.04(4.32–5.89)	2.82 (2.51–3.16)	2.96 (2.64–3.32)
Age				
40–44	1	1	1	1
45–49	0.98(0.79–1.22)	1.10(0.88–1.38)	0.96(0.8–1.15)	1.04 (0.86–1.23)
50–54	0.9(0.71–1.13)	1.10(0.86–1.4)	0.87(0.71–1.07)	0.99 (0.81–1.21)
55–59	1.07(0.85–1.36)	1.41(1.11–1.79)	1.03(0.84–1.26)	1.23 (1.00–1.52)
60–64	0.99(0.75–1.3)	1.17(0.86–1.59)	1.05(0.83–1.32)	1.31 (1.02–1.67)
**Educational level**				
Illiterate	1		1	
Primary school	0.81 (0.62–1.06)		0.94 (0.73–1.2)	
Guidance School	0.81 (0.55–1.20)		0.98 (0.69–1.39)	
High school	0.83 (0.60–1.14)		1.07 (0.81–1.42)	
College	0.42 (0.29–0.6)		0.75 (0.56–1.01)	
**Economic status**				
Low	1		1	1
Medium	1.06 (0.85–1.32)		1.15 (0.95–1.39)	1.24 (1.03–1.49)
High	0.95 (0.78–1.15)		1.14 (0.95–1.36)	1.33 (1.12–1.57)
**Marital status**				
Single	1		1	
Married	1.66 (0.78–3.54)		1.46 (0.79–2.68)	
Widow	2.94 (1.30–6.62)		2.07 (1.08–3.99)	
Divorced	4.09 (1.15–14.56)		2.97 (1.01–8.77)	

* In obese cases, the odds ratio was calculated for people with BMI<25

** In those with BMI≥25, the odds ratio was calculated for people with BMI<25

CI: confidence interval

### Overweight and obesity (BMI≥25)

In the studied sample, 76.2% (95% CI: 75.1–77.4) had a BMI ≥25; the prevalence was 84.2% (95% CI: 83.0–85.4) in women and 65.5% (95% CI: 63.5–67.4) in men (*P*<0.001). Table 1 summarizes the prevalence of BMI ≥25 by age and gender. Age had no significant relationship with BMI ≥ 25 (*P*=0.474). The prevalence of BMI ≥25 showed no significant correlation with education in the simple model (*P*=0.071). There was no difference between the economic groups in terms of overweight and obesity prevalence (*P*=0.293). However, marital status had a significant relationship with the prevalence of BMI ≥25 *(P*=0.018); the prevalence was lower among single people compared to the widowed and divorced. The relationship of BMI ≥25 with studied variables in a multiple logistic regression models showed that female gender (OR=2.96), age greater than 54 yr, and better economic status were among factors associated with BMI ≥25.

## Discussion

Fortunately, regular BMI data collection by the WHO has led to the availability of detailed information on the status of overweight and obesity worldwide. One of the important parts of our study is presenting the prevalence of obesity. Given the completeness of WHO data, we used this information as a basis for some of our comparisons. However, WHO has examined information over a wider age range than our study sample, especially data includes 20 to 40 yr old people, it appears that our overall conclusion about the situation with overweight and obesity should not be based only on this data, thus we tried to show our results with studies on age groups close to ours.

Based on our findings, 3 out of every 4 people were obese or overweight, and 31% were obese; this rate was high in comparison with other studies conducted in Iran. The prevalence of over-weight and obesity was higher in this study compared to the same age groups in the national surveys (9), a study in Shiraz (10), a study in northern Iran (11) and a study in Semnan Province (12). A growing trend was shown for overweight and obesity (7). A high prevalence of overweight and obesity was expected in our study, and these findings indicate that obesity is still on the rise in the Iranian population.

Based on WHO data, the highest prevalence of obesity has been observed in the region of America in people older than 20 yr (26.7%) and the lowest was in South-East Asia (2.7%). Regarding overweight, these regions again show the highest and lowest prevalence rates, respectively. The findings of our study are considerably higher than these regions.

In studies in China (13), Portugal (14), Italy (15), Tanzania (16), and Barbados (17), in the same age group as ours, the prevalence of overweight and obesity was less than in our study. On the contrary, in Syria (18) and Saudi Arabia (19), the prevalence of obesity was higher than ours. The differences observed between these findings may have several causes; economic status and education level are important factors that may partly explain such diversity. In any case, the prevalence of overweight and obesity in this age group may be associated with the epidemic of diseases such as atherosclerosis and diabetes type 2. The findings of this study demonstrated that obesity and overweight are one of the most important health issues in the present population, and control interventions are required.

According to our findings, overweight, especially obesity was significantly more common among women. The rate of obesity was significantly higher in women in the studies in Shiraz (10), northern Iran (11) and Semnan (12) as well. Obesity was also more common in women in Syria (18), Pakistan (20), Tanzania (16), and Nigeria (21); however, in Taiwan (22) the rate of obesity was higher in men. According to WHO data in 2008 (3), 151 of 189 countries showed a higher prevalence of obesity in women than in men; prevalence rates were the same for both genders in 3 countries, and the prevalence of obesity was higher in men in 35 countries. From a public health point of view, special attention must be given to women. Obesity in women can be explained by two causes, 1- social living conditions, 2- physiologic conditions. In most regions of the world, especially Iran, women are less physically active than men (23–24). However, the percentage accumulation of subcutaneous fat in women increases with age as sex hormones decrease. In addition, because of anatomic reasons, the amount of subcutaneous fat is greater in all ages in women compared to men.

Our findings significantly indicated that the prevalence of BMI ≥25 was lower in high and middle economic groups compared to the low economic status group. On the other hand, the observed prevalence of obesity was lower in the high economic group, but this was not significant in the multiple regression models and in the presence of other variables. Our results are consistent with a review (25), concluded that in developing countries, obesity is no longer a disease of high economic group, and the trend of obesity is moving towards low economic status populations. Similar to the results of our study, in another study in Syria (18) obesity was more prevalent is in the low economic group while overweight was seen more commonly in the high economic status group. However, these two observations had no statistical significance, and they studied the economic status, while we only reviewed the economic status. In other studies, obesity is more prevalent in people with high economic status (21) and higher average family income (26).

Our results showed that mean BMI in the high economic status group was lower. However, the difference was not significant in the multiple models. WHO reports (27) suggested that the average BMI in the middle-income countries was higher than that in poor countries of the Eastern Mediterranean region. In our study, one of the causes of an almost equal average BMI in different economic groups can be the relatively high prevalence of overweight and obesity and taking a specific age group (40–64 yr). An increased prevalence of obesity was associated with its decreased inequality, and various groups in society are affected more uniformly (28–29). Overweight and obesity exist with considerable prevalence in all economic groups in urban Iranian populations. Similar to the global trend, the load of the burden is shifting towards lower economic groups, and the impact of factors such as literacy should not be ignored.

## Conclusion

This study provides valuable information concerning overweight and obesity in a middle-aged Iranian population. The prevalence of overweight and obesity in this study was unexpectedly higher than previous studies conducted in Iran. Over-weight and obesity are clearly a more serious problem among women, which is cause for concern. The obesity epidemic, especially in the middle age groups, may lead to an unexpected increase in heart disease, diabetes, and even cancers over the next decade.

## Ethical considerations

Ethical issues (Including plagiarism, informed consent, misconduct, data fabrication and/or falsification, double publication and/or submission, redundancy, etc.) have been completely observed by the authors.
